# Bumetanide Suppression of Angiogenesis in a Rat Model of Oxygen-Induced Retinopathy

**DOI:** 10.3390/ijms21030987

**Published:** 2020-02-02

**Authors:** Sibel Guzel, Charles L. Cai, Taimur Ahmad, Michelle Quan, Gloria B. Valencia, Jacob V. Aranda, Kay D. Beharry

**Affiliations:** 1 Department of Pediatrics, Division of Neonatal-Perinatal Medicine, State University of New York, Downstate Medical Center, Brooklyn, NY 11203, USA; sibelgzel@yahoo.com (S.G.); Charles.cai@downstate.edu (C.L.C.); taahmad00@gmail.com (T.A.); mquanmd@gmail.com (M.Q.); Gloria.valencia@downstate.edu (G.B.V.); Jacob.aranda@downstate.edu (J.V.A.); 2 Department of Ophthalmology, State University of New York, Downstate Medical Center, Brooklyn, NY 11203, USA; 3 State University of New York Eye Institute, New York, NY 10075, USA

**Keywords:** angiogenesis, aquaporins, bumetanide, insulin-like growth factor-I, intermittent hypoxia, retina, vascular endothelial growth factor

## Abstract

Aquaporins (AQPs) are involved in hypoxia-induced angiogenesis and retinal damage. Bumetanide is a diuretic agent, Na^+^/K^+^/Cl^−^ cotransporter (NKCC1), and AQP 1–4 inhibitor. We tested the hypothesis that early postnatal treatment with bumetanide suppresses biomarkers of angiogenesis and decreases severe retinopathy oxygen-induced retinopathy (OIR). Neonatal rats were exposed at birth (P0) to either (1) room air (RA); (2) hyperoxia (50% O_2_); or (3) intermittent hypoxia (IH) consisting of 50% O_2_ with brief, clustered episodes of 12% O_2_ from P0 to postnatal day 14 (P14), during which they were treated intraperitoneally (IP) with bumetanide (0.1 mg/kg/day) or an equivalent volume of saline, on P0–P2. Pups were examined at P14 or allowed to recover in RA from P14–P21. Retinal angiogenesis, morphometry, pathology, AQPs, and angiogenesis biomarkers were determined at P14 and P21. Bumetanide reduced vascular abnormalities associated with severe OIR. This was associated with reductions in AQP-4 and VEGF. Bumetanide suppressed sVEGFR-1 in the serum and vitreous fluid, but levels were increased in the ocular tissues during recovery. Similar responses were noted for IGF-I. In this model, early systemic bumetanide administration reduces severe OIR, the benefits of which appear to be mediated via suppression of AQP-4 and VEGF. Further studies are needed to determine whether bumetanide at the right doses may be considered a potential pharmacologic agent to treat retinal neovascularization.

## 1. Introduction

Retinopathy of prematurity (ROP) has become one of the leading causes of childhood blindness world-wide, with increases in preterm births [1]. In the United States of America (USA), about 14% of childhood blindness is attributed to ROP and in some developing nations, estimates are more than 20% [2]. Studies showed even when oxygen is regulated, ROP can still occur in the smallest and youngest preterm infants [3,4,5]. ROP is a neovascular disease that is characterized by aberrant retinal vascular development. While the pathogenesis is multifactorial, exposure to supraphysiological levels of oxygen and extreme prematurity are major risk factors. Development of ROP appears to occur in two phases. Phase 1 occurs during hyperoxia, which results in suppression of retinal vascularization or in vaso-obliteration. This is due to the suppressive effect of oxygen on vascular endothelial growth factor (VEGF), the most potent mitogen specific to vascular endothelial cells. Phase 2 occurs during recovery from hyperoxia, which results in vaso-proliferation, partly as a result of elevation of VEGF and other growth factors, such as insulin-like growth factor (IGF)-I [6]. Fluctuations in oxygenation have been previously implicated in the development of ROP, based primarily on intermittent arterial sampling [7]. Infants with severe ROP have a higher incidence of intermittent hypoxia (IH) events of longer, more variable, less predictable duration [8,9]. Other risk factors for severe ROP include low birthweight, poor growth, and oxidative distress [10,11,12,13]. Current treatments for severe ROP include laser, preferable to cryotherapy, with 75% success rates [14,15], and targeting VEGF despite long-term adverse outcomes [16]. VEGF is an important angiogenic factor in both physiologic and pathologic retinal vascular development, as well as neurogenesis and organogenesis. The use of anti-VEGF therapies in preterm infants at a critical stage of development may have severe long-term adverse effects [17].

As a delicate extension of the brain and consisting of a complex layer of neural and glial elements, the retina has an extremely high energy demand and metabolic turnover, which generates a need to prevent excess water accumulation during neuronal activity [18]. Ion channels and aquaporins (AQPs) in retinal glial and retinal pigment epithelium (RPE) cells play important roles in maintaining water and electrolyte balance [19]. Among the 13 AQPs, nine (AQP0, AQP1, AQP3, AQP4, APQ5, AQP6, AQP7, AQP9, and AQP11) are found in human and/or rodent retina [20,21,22]. Specifically, AQP1 is mostly expressed in the amacrine cells and the photoreceptor cells and has a polarized distribution in the RPE [20]. Overall expression of AQP1 in the retina is quite low compared to that in the choroid and sclera [21]. AQP4 is predominantly expressed in Müller glial cells and astrocytes in the retina. AQP4 expression in Müller cells has polarized distribution with predominant expression in the end feet membranes, facing the vitreous body of the capillary endothelium. Evidence supports that AQP4 is involved in retinal swelling, and its deletion was shown to be protective for the retina in a transient ischemia-reperfusion model [19]. Given their vital roles in maintaining ocular function, AQPs represent potential targets for future therapeutic development. AQPs have been studied in many ocular diseases such as glaucoma, diabetic retinopathy, and neuromyelitis optica, but there are limited studies on oxygen-induced retinopathy (OIR), particularly IH-induced.

Bumetanide is a loop diuretic shown to have anti-convulsant effects in full-term neonates with hypoxic ischemic encephalopathy and seizures who do not respond to phenobarbital [22,23,24,25]. Bumetanide effects are mediated via its inhibition of the Na^+^K^+^Cl_2_ cotransporter isoform 1 (NKCC1), which transports Cl_2_ into the cell, and the K^+^Cl_2_ cotransporter isoform 2 (KCC2) that moves Cl_2_ out of the cell [26]. During brain and retinal development, the action of GABA on neurons shifts from excitatory to inhibitory due to developmental changes in NKCC1 and KCC2 [27]. Bumetanide also inhibits AQP1 and AQP4 [28]. Simultaneous inhibition of NKCC1 and AQP4 with bumetanide reduced edema and spinal cord tissue destruction in rats [29]. Despite the important role of neonatal IH, as well as AQPs, in the pathogenesis of ROP, there are no studies examining the potential therapeutic benefit of bumetanide in IH-induced OIR. Therefore, the goal of this study was to test the hypothesis that early postnatal treatment with bumetanide suppresses biomarkers of angiogenesis and decreases the severity of OIR in our rat model of neonatal IH [30,31,32,33,34].

## 2. Results

### 2.1. Effect of Bumetanide on Eye Opening

Eye opening in rats (also known as the caecal period—the time from conception to eye opening) occurs at or around P14, coinciding with maturation of the retinal neural circuitry. Table 1 shows that bumetanide improved the caecal period in over 80% of rats in RA and over 75% in IH. No difference was noted with hyperoxia exposure.

### 2.2. Effect of Bumetanide on Growth

Percentage change in body weight and linear growth from P0 is presented in Table 2. At P14, exposure to hyperoxia with bumetanide and placebo saline resulted in higher weight accretion, although body length was decreased. Bumetanide decreased body weight but increased body length with treatment in IH. Animals exposed to IH and treated with placebo saline also were growth suppressed compared to their RA and hyperoxia-exposed littermates.

### 2.3. Effect of Bumetanide on AQPs

**Figure 1:**
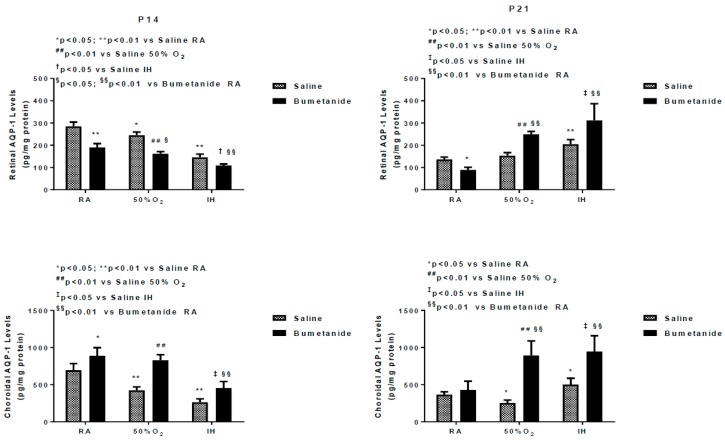
Effect of early postnatal bumetanide on aquaporin (AQP)-1 in the retina and choroid of neonatal rats at postnatal day 14 (P14) and P21. Newborn rats received a single daily intraperitoneal (IP) dose of either bumetanide (0.1 mg/kg/day) or an equivalent volume on saline on the day of birth (P0) and at P1 and P2. Pups with their dams were randomized to either room air (RA), hyperoxia (50% O_2_), or intermittent hypoxia (IH). Data are presented as the mean ± SEM (*n* = 4 samples/group). During treatment, bumetanide decreased AQP1 in the retina, but not in the choroid.

Figure 1 represents the AQP1 and AQP4 levels in the retinal and choroidal homogenates at P14 and P21. At P14, bumetanide significantly decreased retinal AQP1 levels under all oxygen conditions in the retina, while levels were increased in the choroid.

**Figure 2:**
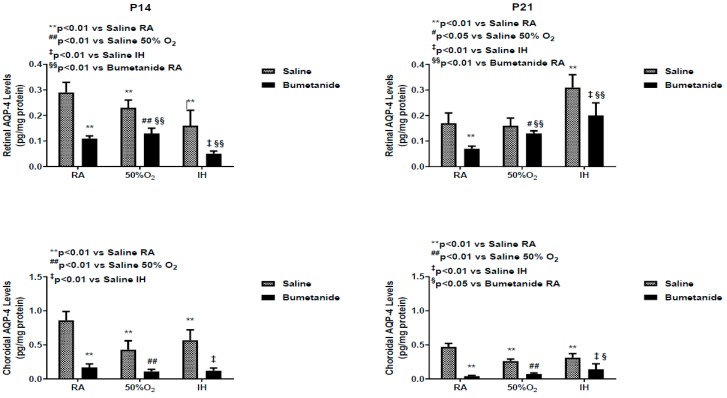
Effect of early postnatal bumetanide on aquaporin (AQP)-4 in the retina and choroid of neonatal rats at postnatal day 14 (P14) and P21. Bumetanide decreased AQP4 in the retina and choroid during treatment and recovery/re-oxygenation. Groups are as described in Figure 1. Data are presented as the mean ± SEM (*n* = 4 samples/group). RA (room air); O_2_ (oxygen); IH (intermittent hypoxia).

At P21, there was a rebound elevation in the retinas exposed to hyperoxia and IH, but not in RA. In the choroid, the levels further increased in the retinas exposed to hyperoxia and IH. In contrast, bumetanide suppressed AQP4 levels in the retina and choroid exposed to all oxygen conditions at P14, an effect that remained sustained at P21 (Figure 2).

### 2.4. Effect of Bumetanide on VEGF

**Figure 3:**
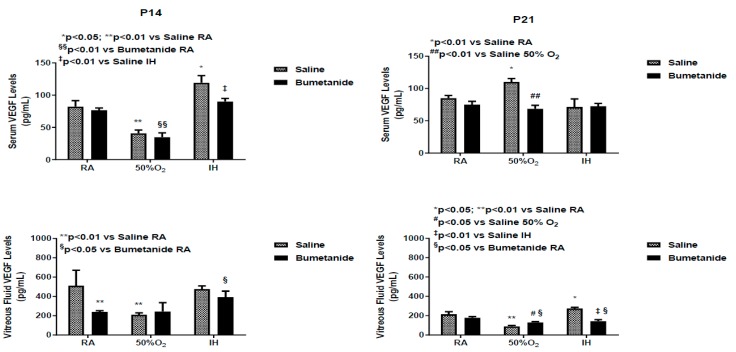
Effect of early postnatal bumetanide on vascular endothelial growth factor (VEGF) levels in the serum (*n* = 6 samples/group) and vitreous fluid (*n* = 4 samples/group) of neonatal rats at postnatal day 14 (P14) and P21. Bumetanide suppressed serum and vitreous VEGF in IH. Groups are as described in Figure 1. Data are presented as the mean ± SEM. RA (room air); O_2_ (oxygen); IH (intermittent hypoxia).

Studies have shown that the vitreous fluid is a reservoir for growth factors, and levels are higher compared to serum, suggesting local production [35]. Figure 3 demonstrates differences between serum and vitreous fluid levels of VEGF at P14 and P21 in response to bumetanide treatment in hyperoxia and IH. Indeed, levels of VEGF were higher than those in serum. At P14, serum levels of VEGF were comparable in RA and hyperoxia, although, as expected, serum levels declined in the groups exposed to hyperoxia compared to RA. In IH, VEGF levels increased in both treated groups, but the levels were lower with bumetanide compared to saline treatment in IH, but not in RA. Bumetanide suppressed VEGF levels in the vitreous fluid in RA. Hyperoxia decreased VEGF levels in the vitreous fluid in both treated groups. Bumetanide reduced VEGF levels in IH compared to placebo saline, but not compared to treatment in RA. At P21, serum levels increased in the saline group exposed to hyperoxia and were lower in the bumetanide-treated group. In the vitreous fluid, VEGF levels remained suppressed in the groups exposed to hyperoxia and were elevated in the placebo saline group exposed to IH. Treatment with bumetanide suppressed VEGF levels in IH.

**Figure 4:**
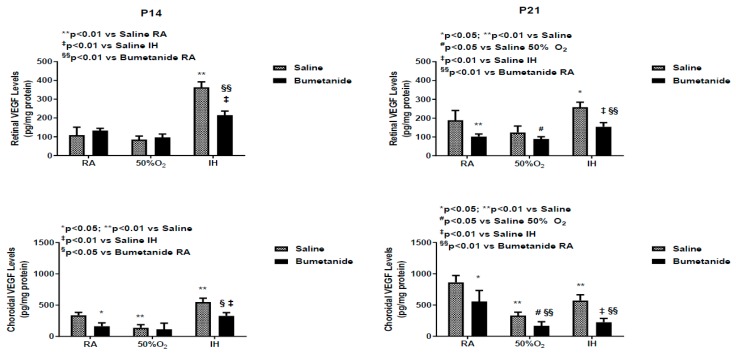
Effect of early postnatal bumetanide on vascular endothelial growth factor (VEGF) levels in the retina and choroid of neonatal rats at postnatal day 14 (P14) and P21. Bumetanide suppressed retinal and choroidal VEGF in IH. Groups are as described in Figure 1. Data are presented as the mean ± SEM (*n* = 4 samples/group). RA (room air); O_2_ (oxygen); IH (intermittent hypoxia).

Figure 4 represents VEGF levels in the retinal and choroidal homogenates, which were standardized using total cellular protein levels. At P14, VEGF levels were elevated only in the groups exposed to IH and bumetanide was successful in reducing VEGF, but not to RA levels. In the choroid, bumetanide reduced VEGF in RA, levels declined in IH with both treatments, and increased in IH. Similarly, bumetanide reduced choroidal VEGF levels but not to the levels comparable with RA.

### 2.5. Effect of Bumetanide on sVEGFR-1

**Figure 5:**
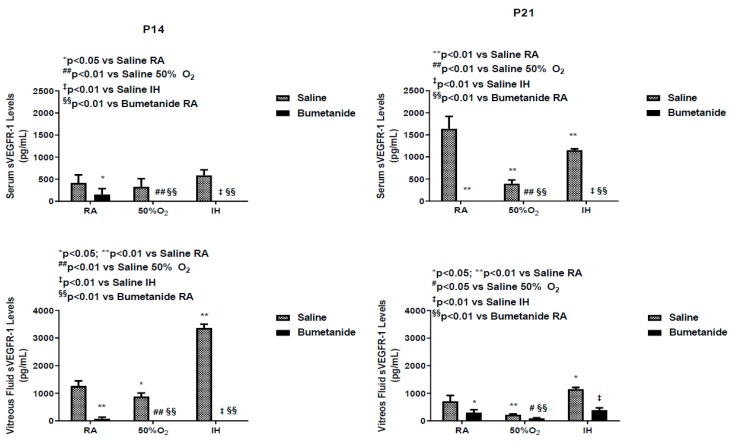
Effect of early postnatal bumetanide on soluble vascular endothelial growth factor receptor (sVEGFR)-1 levels in the serum (*n* = 6 samples/group) and vitreous fluid (*n* = 4 samples/group) of neonatal rats at postnatal day 14 (P14) and P21. Bumetanide markedly suppressed serum and vitreous sVEGFR-1. Groups are as described in Figure 1. Data are presented as the mean ± SEM. RA (room air); O_2_ (oxygen); IH (intermittent hypoxia).

Soluble VEGF receptor (sVEGFR)-1 is a splice variant of the membrane type receptor. It prevents VEGF action by competitively binding with VEGF, making it unavailable to membrane VEGFR-1. Figure 5 represents sVEGFR-1 levels in the serum and vitreous fluid at P14 and P21. At P14, serum sVEGFR-1 levels were relatively low in all oxygen environments and were significantly suppressed with bumetanide. In contrast, vitreous fluid levels were 2- to 3-fold higher and were suppressed with bumetanide in all oxygen environments. At P21, serum levels were higher in all untreated groups with the lowest concentrations measured in hyperoxia. sVEGFR-1 levels remained suppressed in the bumetanide groups. In the vitreous fluid, levels were lower in the untreated groups compared to levels at P14 and remained suppressed in the groups treated with bumetanide. 

**Figure 6:**
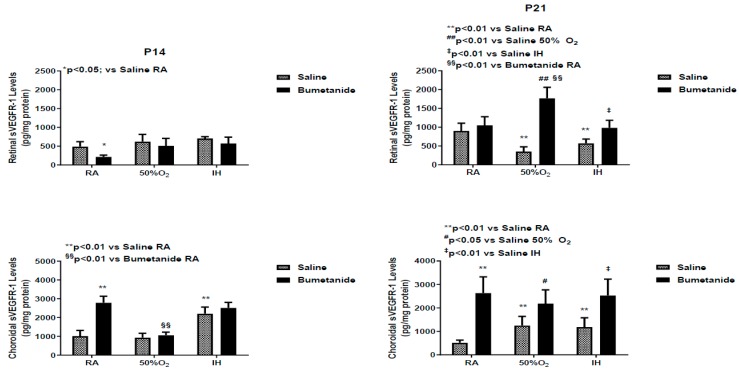
Effect of early postnatal bumetanide on soluble vascular endothelial growth factor receptor (sVEGFR)-1 levels in the retina and choroid of neonatal rats at postnatal day 14 (P14) and P21. Bumetanide was not effective for suppression of retinal and choroidal sVEGFR-1. Groups are as described in Figure 1. Data are presented as the mean ± SEM (*n* = 4 samples/group). RA (room air); O_2_ (oxygen); IH (intermittent hypoxia).

sVEGFR-1 levels in the retina and choroidal homogenates at P14 and P21 are presented in Figure 6. The data show that at P14, bumetanide suppressed retinal sVEGFR-1 levels in RA only. An opposite effect was seen in the choroid. Exposure to hyperoxia and bumetanide reduced choroidal sVEGFR-1 levels, while exposure to IH increased the levels in both treatment groups. At P21, retinal levels were lowest in the saline-treated groups exposed to hyperoxia and IH. Treatment with bumetanide in hyperoxia and IH resulted in elevated levels. In the choroid, sVEGFR-1 levels increased in the saline-treated groups exposed to hyperoxia and IH and in all bumetanide-treated groups. 

### 2.6. Effect of Bumetanide on IGF-1 Levels

**Figure 7:**
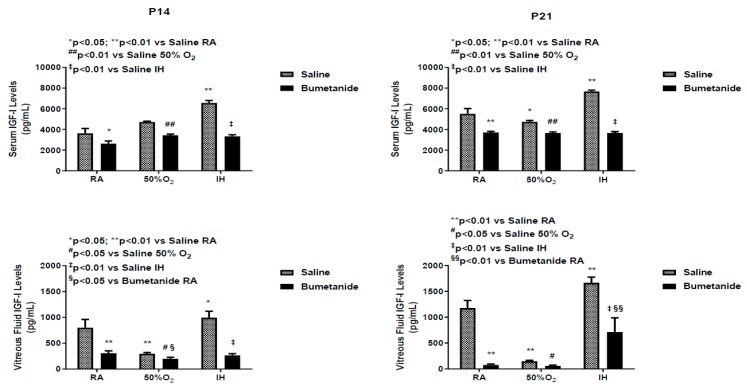
Effect of early postnatal bumetanide on insulin-like growth factor (IGF)-I levels in the serum (*n* = 6 samples/group) and vitreous fluid (*n* = 4 samples/group) of neonatal rats at postnatal day 14 (P14) and P21. Bumetanide conferred a sustained suppressive effect on serum and vitreous IGF-1. Groups are as described in Figure 1. Data are the presented as mean ± SEM. RA (room air); O_2_ (oxygen); IH (intermittent hypoxia).

IGF-I is a permissive factor for VEGF, and serum levels have been shown to be deficient in preterm infants who developed severe ROP [6,36,37]. Serum and vitreous fluid IGF-I levels at P14 and P21 are presented in Figure 7. At P14, serum levels were increased in the placebo group exposed to IH. Bumetanide reduced IGF-I levels in all oxygen environments. Similar findings were noted in the vitreous fluid. At P21, serum levels remained elevated in the saline-treated group exposed to IH and suppressed with bumetanide treatment. Vitreous fluid IGF-I levels also remained elevated in the saline-treated groups exposed to IH, but declined substantially in the bumetanide-treated groups exposed to RA and hyperoxia. Although levels were lower than in the control, they remained higher than levels in RA and hyperoxia. 

**Figure 8:**
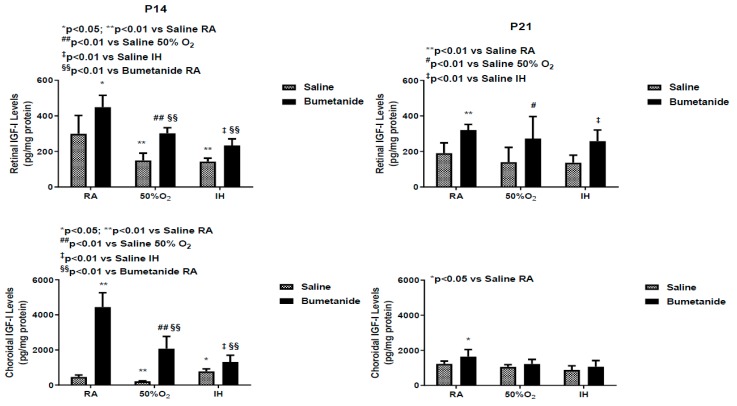
Effect of early postnatal bumetanide on insulin-like growth factor (IGF)-I levels in the retina and choroid of neonatal rats at postnatal day 14 (P14) and P21. Bumetanide increased retinal and choroidal IGF-1. Groups are as described in Figure 1. Data are presented as the mean ± SEM (*n* = 4 samples/group). RA (room air); O_2_ (oxygen); IH (intermittent hypoxia).

Figure 8 represents the IGF-I levels in the retina and choroid at P14 and P21. Exposure to hyperoxia and IH reduced retinal IGF-I despite treatment, but the levels were lower in the placebo saline groups. Similar findings occurred in the choroid. At P21, retinal IGF-I levels remained elevated in all bumetanide groups. Choroidal levels were elevated only in the bumetanide-treated group exposed to RA. 

### 2.7. Effect of Bumetanide on Retinal Vasculature

**Figure 9:**
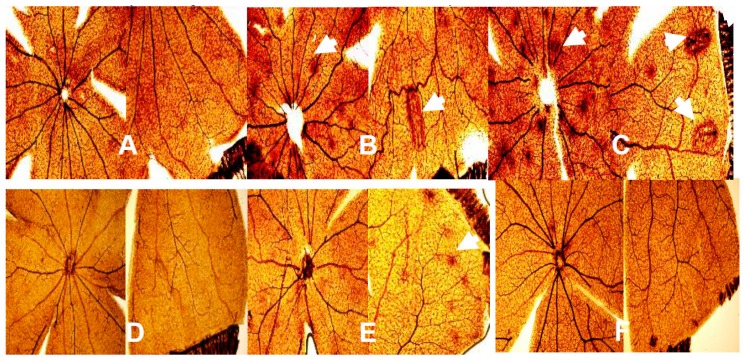
Representative retinal adenosine diphosphatase (ADPase)-stained flatmounts for retinas from neonatal rats at postnatal day 21 (P21). The upper panels are the saline-treated groups and the lower panels are the bumetanide-treated groups. The RA-exposed retinas are panels (**A**) and (**D**); the hyperoxia-exposed retinas are panels (**B**) and (**E**); and the IH-exposed retinas are panels (**C**) and (**F**). In each composite panel, the left side corresponds to the optic disk and the right side to the periphery. Images were captured at 10× magnification. Exposure to hyperoxia and IH caused vascular tortuosity and punctate hemorrhages (arrow) at the optical disk and periphery. Bumetanide improved retinal vascular abnormalities but did not prevent it, particularly in hyperoxia.

Figure 9 shows representative images of the retinal flatmounts stained with ADPase. The upper panels are the saline-treated groups and the lower panels are the bumetanide-treated groups. The RA-exposed retinas are panels A and D; the hyperoxia-exposed retinas are panels B and E; and the IH-exposed retinas are panels C and F. In each composite panel, the left side corresponds to the optic disk and the right side to the periphery. In room air, bumetanide treatment resulted in less vascular abundance and capillary dropout in the optic disk and periphery (panel D) compared to saline controls (panel A). Hyperoxia resulted in many punctate hemorrhages around the optic disk (arrow) and in vascular tortuosity and large hemorrhages at the periphery (arrows) in the saline-treated groups (panel B). Bumetanide treatment in hyperoxia (panel E) reduced the occurrences of hemorrhage at the optic disk and vascular tortuosity at the periphery, but hemorrhages remained at the periphery (arrow). Exposure to IH caused an abundance of hemorrhage and vascular tortuosity at the optic disk (arrows), as well as large hemorrhages and enlarged tortuous vessels at the periphery (arrows) in the placebo saline group (panel C). In contrast, exposure to IH with bumetanide treatment resulted in significantly less hemorrhages and vessel tortuosity at the optic disk and periphery, although there was some evidence of capillary dropout (panel F).

### 2.8. Effect of Bumetanide on Retinal Layers

**Figure 10:**
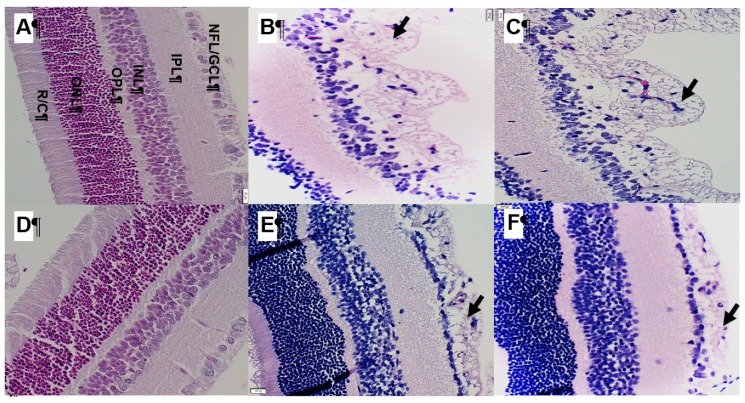
Representative images of the hematoxylin and eosin (H&E) stained retinal layers. The upper panels are the saline-treated groups exposed to room air (RA) (**A**), hyperoxia (**B**) or intermittent hypoxia (IH) (**C**), and the lower panels are the bumetanide-treated groups exposed to RA (**D**), hyperoxia (**E**) or IH (**F**). The layers are labeled in panel A. Images were captured at 40× magnification (scale bar is 20 µm for each panel). Exposure to hyperoxia and IH increased the number of endothelial cells violating the vitreous fluid and widened the nerve fiber layer (NFL)/ganglion cell layer (GCL) (arrows). Bumetanide improved retinal thickness, decreased the number of migrating endothelial cells, and reduced the NFL/GCL layer, but the values were not consistent with controls.

Figure 10 shows representative images of the H&E stained retinal layers. The upper panels are the saline-treated groups exposed to RA (Figure 10A), hyperoxia (Figure 10B) or IH (Figure 10C), and the lower panels are the bumetanide-treated groups exposed to RA (Figure 10D), hyperoxia (Figure 10E) or IH (Figure 10F). The layers are labeled in panel A. The RA-exposed retinas appear normal with all layers intact. In contrast, retinas exposed to hyperoxia (Figure 10B,E) showed widening of the NFL/GCL layer with an abundance of retinal endothelial cells migrating into the vitreous fluid (arrows). Exposure to IH with saline treatment (Figure 10C) showed worsening of the NFL/GCL layers with cells violating the vitreous fluid (arrow). Bumetanide treatment in IH significantly reduced the NFL/GCL layer and the number of cells migrating into the vitreous fluid (Figure 10F).

### 2.9. Effect of Bumetanide on Retinal Morphometry

Table 3 shows retinal morphometric analyses at P21. The data show that exposure to hyperoxia and IH resulted in significant vessel tortuosity during the recovery period, an effect that was curtailed with bumetanide. Hyperoxia moderately increased arterial diameter, while IH decreased it. In contrast, hyperoxia decreased venous diameter. Bumetanide reversed the effect in hyperoxia only. The number of endothelial cells in the NFL/GCL layer increased substantially in all groups exposed to hyperoxia and IH. While bumetanide significantly reduced the numbers, control levels were not achieved. Furthermore, treatment with bumetanide in RA also resulted in higher cell numbers. Overall retinal thickness was also higher in all groups exposed to hyperoxia and IH, although treatment with bumetanide decreased the retinal thickness. This finding was consistent in each of the retinal layers, except for IPL thickness in IH, which showed no difference from bumetanide treatment. While bumetanide treatment attenuated the effects of hyperoxia and IH during recovery, it was not effective for reversing the outcomes.

## 3. Discussion

The goal of this study was to test the hypothesis that early postnatal bumetanide suppresses biomarkers of angiogenesis and decreases the severity of IH-induced OIR. The dose was based on a clinical trial in human neonates with hypoxic ischemic encephalopathy [38]. The major findings are as follows: (1) Both AQP1 and AQP4 decreased in the ocular compartment during exposure to hyperoxia and IH but over-expressed during recovery. These findings suggest that AQP1 and AQP4 may play a key role in the restorative process following hyperoxia/hypoxia. This phase is usually associated with neovascularization due to restitution of blood flow through damaged vessels, overproduction of VEGF, and other growth factors. Studies show that exposure of AQP4 knockout mice to hypoxia results in reduced retinal neovascularization, demonstrating the key role of AQP4 in this process [39]; (2) Bumetanide appears to be a more potent and consistent inhibitor of ocular AQP4 than it is for AQP1, at least in the setting of neonatal IH. The significance of this finding is that AQP4, which is detected primarily in the blood vessels, astrocytes, and Müller cells, is involved in water flux from the blood vessels to the retina and retinal swelling [40,41], a major characteristic of retinopathy [42]. Bumetanide may reduce retinal swelling, as well as astroglial swelling and loss in retinal injury. This is corroborated by the reduction in retinal thickness presented in Table 3; (3) Choroidal levels of AQP-1 were found to be higher than the retinal levels in all groups. AQP1 has been shown to be expressed in the choroid, which provides blood supply to the avascular retina. AQP1 is also high in hyaloid vessels, a vascular system that supplies blood to the retina and lens during early eye development [43]. AQP1 is also found in retinal endothelial cells but only in a small percentage of newly formed retinal vessels [44]. 

Confirming previous studies, our data suggest that AQPs can be targeted in retinal proliferative diseases. Bumetanide suppressed the retinal AQP1 levels up to 14 days, but this suppression could not be sustained up to 21 days, and there was a rebound increase at P21. This rebound happened earlier at P14 in the choroid compared to 21 days in the retina. Elevations in AQP1 during the re-oxygenation/reperfusion phase may be due to a switch from AQP4. It is well known that ischemia–reperfusion causes alteration of AQP1 and AQP4 in the retina [45,46]. A similar switch has been reported in ocular inflammation, diabetic retinopathy, and ocular hypertension [47]; (4) Bumetanide decreased IH-induced elevations in VEGF in the ocular compartment. Studies show a relationship between AQP4 and hypoxia-induced VEGF upregulation in a mouse model of OIR. Retinal VEGF levels were not elevated in AQP4 knockout mice exposed to hypoxia, an effect that may occur via reduced accessibility of HIF_1α_ to the VEGF promoter [39]. Other studies demonstrated that Müller cell swelling was mediated by VEGF and AQP4 [40], further demonstrating a link between VEGF and AQP4; (5) Bumetanide reductions in VEGF were associated with reductions in the severity of OIR, particularly hemorrhages, vascular tortuosity, and migration of retinal endothelial cells into the vitreous fluid, characteristics that are highly influenced by VEGF and AQP4. Studies show robust AQP4 expression in rat retinas injured by high blast exposures. In those studies, glial fibrillary acidic protein (GFAP) staining confirmed that AQP4-postiive cells were astrocytes that are confined to the nerve fiber layer (NFL)/ganglion cell layer (GCL) layer [48]. This raises the possibility that AQP4, together with VEGF colocalization, is highly involved in the migration and invasion of retinal endothelial cells into the vitreous fluid; (6) Bumetanide is a potent suppresser of sVEGFR-1 in serum and vitreous fluid. Studies show that sVEGFR-1 is a VEGF-induced angiogenesis inhibitor that binds to VEGF with high affinity [49]. Levels in the vitreous fluid correlate significantly with plasma levels in patients with proliferative retinopathy [50] and are increased by high levels of VEGF, which may be a compensatory mechanism to curtail VEGF over-production. The suppressive effects on sVEGFR-1 may be secondary to bumetanide reduction of VEGF resulting in less vascular abundance. Nevertheless, we do not know for certain if the decreased VEGF and sVEGFR-1 is a consequence of bumetanide or increased binding of sVEGFR-1 to VEGF; and (7) Bumetanide decreases serum and vitreous fluid IGF-I, but not in the retina and choroid. In addition to its permissive relationship with VEGF, IGF-I has been shown to stimulate the bumetanide-sensitive Na^+^/K^+^/Cl^-^ cotransporter (NKCC1) as part of its mitogen signal, which is inhibited by bumetanide [51]. Conversely, IGF-I appears to have similar effects on neuroexcitation. Studies show that IGF-I decreased the NKCC1/KCC2 ratio, favoring a developmental switch of GABAergic responses from excitation to inhibition in the visual cortex, contributing to precocious development of visual acuity [52]. In our studies, both retinal and choroidal IGF-I levels were elevated with bumetanide in all groups which may confirm the beneficial effects of bumetanide. 

Taken together, the results of these experiments confirm previous findings and demonstrate that bumetanide has not only anti-angiogenic properties in the setting of IH-induced OIR but may also participate in precocious development of visual acuity. This was most certainly demonstrated by eye opening presented in Table 1. The caecal period in rodents (from conception to eye opening) is important as it represents maturation of the retinal neural circuitry, corneal development, and overall maturation of the visual cortex [53,54,55]. At birth, the rat retina is largely undifferentiated, and by P14 (eye opening), many retinal cells are mature. As noted above, precocious eye opening with bumetanide may be linked to elevations in IGF-I and suggests improved visual acuity. IGF-I is also important for postnatal growth. It is well known that preterm infants who are at the highest risk for development of severe ROP are more likely to be growth suppressed. Poor growth and low systemic IGF-I levels have been shown to be important predictors of severe ROP [36,37]. The computerized clinical algorithm Weight, Insulin-like growth factor (IGF-1), Neonatal Retinopathy of Prematurity (WINROP) has been developed as a non-invasive tool for identifying infants at risk for ROP based on postnatal weight gain and IGF-I levels [56]. It was interesting to note that while bumetanide increased IGF-I in the retina and choroid, the levels were decreased in the serum and vitreous fluid (which generally reflects serum levels). These reductions in serum IGF-I may account for the reductions in body weight accretion seen in the hyperoxia and IH groups treated with bumetanide. Regardless, the retinal outcomes were significantly improved. This finding shows that levels in the systemic compartment may not necessarily reflect retinal and choroidal levels. Bumetanide reduction in body weight accretion is likely due to water loss. 

We utilized a well-established IH paradigm simulating IH, experienced by extremely low gestational age neonates who are at the highest risk for severe ROP. The use of neonatal IH models for studying severe ROP has provided key insights into the pathogenesis of the disease. In the neonatal rat, retinal vascularization occurs ex utero. At birth, pups have incompletely vascularized retinas similar to the immature retinal vasculature of the human premature infant [57]. All OIR models have limitations because they use term newborn, instead of premature animals. Even though they complete retinal vascular development after term birth, term newborn animals are healthy and do not have the comorbidities of preterm infants that contribute to ROP. Neonatologists strive to avoid high oxygen in the perinatal period, but most animal models use high oxygen (or hypoxia) for prolonged periods, making them less representative of the human situation. The most representative model of human ROP in the era of oxygen regulation is the rat OIR model, which has aspects of both vaso-obliteration centrally and delayed physiologic retinal vascularization peripherally [58]. The appearance of first delayed physiologic retinal vascular development followed by neovascularization at the junction of vascular and avascular retina at day 18 is similar in appearance to type 1 severe ROP [14]. Typically, the duration of the hyperoxia and hypoxia fluctuations in the rat model is 24 h [58], whereas in the human preterm infant, minute-to-minute fluctuations occur [8,9,59]. Therefore, our OIR model, with shorter and more frequent hypoxic episodes, simulates the experience of preterm babies more realistically than this classic model. Overcrowding the rat pups provided us similar extrauterine growth restriction that premature babies experience, a factor strongly associated with severe ROP.

## 4. Materials and Methods

All experiments were approved by the State University of New York, Downstate Medical Center Institutional Animal Care and Use Committee (Protocol #14-10436; approved December 19, 2014), Brooklyn, NY. Animals were managed according to the Association for Research in Vision and Ophthalmology (ARVO) Statement for the Use of Animals in Ophthalmic and Visual Research.

### 4.1. Experimental Design

Timed-pregnant Sprague Dawley rats were purchased from Charles River Laboratories (Wilmington, MA, USA) and housed under standard conditions with free access to standard diet and water. Newborn rat pups (4 litters per experiment) were pooled to eliminate litter differences and equalize birth weight in each group. Nine male and nine female pups (gender determined by the anogenital distance) were randomly selected and placed with one dam for each group. A litter size of 18 pups was found to be the maximum number of pups that the dam could handle with no mortality. This expanded litter size simulates poor nutrition of ELGANs who develop severe ROP. Each pup was weighed (grams) and measured for linear growth (crown to rump length in centimeters) at birth, P14 and P21. A total of 12 groups of 18 rat pups were studied. Four groups were exposed to room air (RA), four to hyperoxia (50% O_2_), and four to intermittent hypoxia (IH), consisting of 50% O_2_ with brief episodes of 12% O_2_ from birth (P0) to P14, as previously described [32,33,34,60]. Pups were treated intraperitoneally (IP) with either bumetanide (0.1 mg/kg/day) or an equivalent volume of saline at P0, P1, and P2. Pups were studied at P14 for immediate effects of IH or were placed in RA for re-oxygenation/recovery until P21. At P14, the number of animals that opened their eyes was recorded.

### 4.2. Hyperoxia or IH Cycling

Animals randomized to hyperoxia or IH were placed with the dams in specialized oxygen chambers attached to an oxy-cycler (BioSpherix, New York, USA). Animals randomized to hyperoxia were exposed to constant 50% O_2_ from P0–P14, and animals randomized to IH were exposed to 50% O_2_ for 30 min followed by eight IH cycles per day from P0–P14. The IH paradigm was previously described [32,33,34,61] and mimics preterm newborn infants who experience recurrent apnea and desaturations.

### 4.3. Sample Collection and Processing

For serum biomarkers of angiogenesis, whole blood was collected in Eppendorf tubes and placed on ice for 30 min (*n* = 6 samples per group). The samples were centrifuged at 3500 rpm for 20 min at 4 °C. The resulting serum was transferred to a clean Eppendorf tube and frozen at −20 °C until assay for VEGF, sVEGFR-1 and IGF-I. For collection of vitreous fluid, eyes were enucleated and rinsed in ice-cold phosphate buffered saline (PBS, pH 7.4) on ice. The vitreous fluid was collected by first piercing the eyes and placing them in Eppendorf collection tubes. The eyes were centrifuged at 3000 rpm for 20 min. In each group, vitreous fluid samples were pooled for a total of four samples per group. Samples that were contaminated with blood were not used. The retinas and choroids were excised under a dissecting microscope (Olympus America Inc., Center Valley, PA, USA and placed in sterile lysing matrix D tubes (2.0 mL) ceramic spheres (MP Biomedicals, Santa Ana, CA, USA) and 1.0 mL ice-cold sterile PBS prior to snap-freezing in liquid nitrogen. In each group, samples were pooled for a total of four samples per group and were stored at −80 °C until analysis. On the day of analyses, the tubes containing retinas and choroids were homogenized using the FastPrep-24 instrument (MP Biomedicals, Santa Ana, CA, USA), after which they were centrifuged at 4 °C at 10,000 rpm for 20 min. The supernatant was filtered, and the filtrate was used for the assays. For ADPase-stained retinas, whole eyes were placed in 4% paraformaldehyde on ice for 120 min, and the retinas were harvested and flatmounted. For retinal layer integrity, whole eyes were fixed in-situ in 10% neutral-buffered formalin (NBF), then enucleated, marked for orientation, placed in 4% NBF, and processed for H&E staining using standard techniques (Histowiz, Inc., Brooklyn, NY, USA).

### 4.4. Aquaporin Assays

AQP1 and four levels in the retinal and choroidal homogenates were determined using quantitative sandwich rat enzyme-linked immunosorbent assay (ELISA) kits purchased from MyBiosource, Inc. (San Diego, CA, USA), according to the manufacturer’s protocol. The sensitivity of the kit is 0.1 ng/mL, the detection range is 0.25–8 ng/mL, and the intra-and inter-assay coefficients of variation are less than 15%. Data were standardized using total cellular protein levels.

### 4.5. Assay of Angiogenesis Biomarkers

VEGF, sVEGFR-1, and IGF-I levels in the serum, vitreous fluid, and retinal and choroid homogenates were assayed using commercially-available quantikine ELISA kits from R & D Systems (Minneapolis, MN, USA), as previously described [32,33,34]. All assays were conducted according to the manufacturer’s protocol. All tissue data were standardized using total cellular protein levels.

### 4.6. Total Cellular Protein Levels

On the day of the assays, an aliquot (10 µL) of the retinal and choroid homogenates was utilized for total cellular protein levels using the Bradford method (Bio-Rad, Hercules, CA, USA) with bovine serum albumin as a standard.

### 4.7. ADPase Staining of Retinal Flatmounts

Retinal flatmounts were stained following incubation overnight in 4% paraformaldehyde at 4 °C, as previously described [32,33,34]. Images were captured at 20× magnification using an Olympus BX53 microscope (Olympus America Inc., Center Valley, PA, USA), DP72 digital camera (Olympus America Inc., Center Valley, PA, USA), and CellSens imaging software (Olympus America Inc., Center Valley, PA, USA) attached to a Dell Precision T3500 computer (Dell Technologies, Round Rock, Texas, USA).

### 4.8. Retinal Morphometric Analyses

ADPase-stained retinal flatmounts were used to determine the tortuosity index and vessel diameter. For the tortuosity index, a line was traced along the tortuous arteries using the polyline tool compared to a straight line traced from the vessel origin at the optic disk to the branch point using the arbitrary line tool of the CellSens software (Olympus America Inc., Center Valley, PA, USA) as previously described [33,34,60,61]. Vessel diameter at the optic disk was quantified using the arbitrary line tool of the CellSens Dimension software (Olympus America Inc., Center Valley, PA, USA). The number of endothelial cells present in the nerve fiber layer (NFL)/ganglion cell layer (GCL), the total retinal thickness, and the thickness of the NFL/GCL, inner plexiform layer (IPL), INL (inner nuclear layer), and ONL (outer nuclear layer) were quantified in the H&E stained sections using the count and measure of region of interest and arbitrary line tools of CellSens Dimension software (Olympus America Inc., Center Valley, PA, USA).

### 4.9. Statistical Analysis

To determine differences among the group, a test for normality was first conducted using Bartlett’s test. Normally distributed data were analyzed using one-way analysis of variance (ANOVA) with Dunnett’s post-hoc tests for multiple comparisons. Non-normally distributed data were analyzed using the Kruskall–Wallis test with Dunn’s post-hoc multiple comparison test. Data are presented as the mean ± SEM, and a *p*-value of <0.05 was considered as statistically significant, using Statistical Package for Social Sciences (SPSS) version 16.0 (SPSS Inc., Chicago, IL, USA). Graphs were prepared using GraphPad Prizm version 7.03 (GraphPad, San Diego, CA, USA).

## 5. Conclusions

Advances in the treatment of retinal angiogenesis and edema have been rapid. Increased clinical use of anti-VEGF agents curtails vascular permeability, retinal angiogenesis, and intraretinal hemorrhages. However, many issues remain to be investigated in order to optimize treatment. Recent developments in our understanding of the molecular pathogenesis of these conditions and healthy homeostasis have and will continue to result in the generation of more specific and potent therapeutic agents. Due to the complexity of retinal angiogenic and edematous disorders, the existence of a magic bullet drug that fulfils all the criteria for therapeutic success is unlikely. Therefore, understanding the merits and shortcomings of available agents is necessary in order to find the best combination of therapies to achieve the best therapeutic outcome [62]. Another challenge with treating the retina is the difficulty of accessing posteriorly and anteriorly because of the blood–retinal barriers and the diminished therapeutic response to a drug after repeated administration over time [62]. Future studies examining sustained therapeutic action, efficacy/potency, target specificity, and ocular and systemic safety are needed. This work has direct clinical implications for the treatment of ROP. The major findings of this study were that early postnatal bumetanide reduced the severity of OIR induced by neonatal IH likely due to suppression of AQP4 and that bumetanide shortened the caecal period, suggesting precocious retinal neural and visual cortex maturation, corneal development, and possible improved visual acuity. However, despite these benefits, bumetanide did not prevent OIR. More studies are needed to determine the exact dosing regimen at different windows of time. A major challenge is that early and vascular phases of human ROP may not be sufficiently distinct in an individual preterm infant who experiences many IH episodes in order to identify a safe window of treatment opportunity without future adverse events. A delicate dosing balancing is required when inhibiting growth factors (VEGF) and water channels (AQPs) to suspend pathological neovascular progression while maintaining sufficient levels for normal retinal cell survival and visual acuity.

## Figures and Tables

**Table 1:** Eye Opening at P14.

Eye	Saline RA	Bumetanide RA	Saline 50% O_2_	Bumetanide 50% O_2_	Saline IH	Bumetanide IH
Left Eye	21 (58%)	30 (83%) *	22 (61%)	23 (64%)	13 (36%)	31 (86%) **
Right Eye	22 (61%)	33 (92%) **	24 (67%)	23 (64%)	11 (31%)	28 (78%) **
Both Eyes	20 (56%)	29 (81%) *	23 (64%)	22 (61%)	4 (11%)	27 (75%) **

*n* = 36 per group (* *p* < 0.05; ** *p* < 0.01 vs. saline by Fisher’s exact test). RA (room air); O_2_ (oxygen); IH (intermittent hypoxia).

**Table 2:** Growth.

% Change from P0	Saline RA	Bumetanide RA	Saline 50% O_2_	Bumetanide 50% O_2_	Saline IH	Bumetanide IH
**P14 (*n* = 18/group):**						
Weight (g)	229.5 ± 12.2	216.7 ± 8.2	306.4 ± 8.7 **	255.6 ± 11.5 ^§§^	263.6 ± 11.4	232.1 ± 6.0 ^†^
Length (cm)	61.3 ± 2.2	62.0 ± 3.6	40.3 ± 1.5 **	55.0 ± 2.4 ^##^	43.8 ± 1.6	54.1 ± 2.2 ^‡^
**P21 (*n* = 18/group):**						
Weight (g)	444.7 ± 13.4	474.5 ± 19.7	456.9 ± 13.4	268.1 ± 10.1 ^##,§§^	376.0 ± 17.6 **	233.0 ± 7.6 ^§§,‡^
Length (cm)	86.3 ± 4.2	100.9 ± 3.1 **	100.5 ± 1.2 **	64.1 ± 2.6 ^##,§§^	89.6 ± 2.7	59.6 ± 2.1 ^§§,‡^

Data are the mean ± SEM (* *p* < 0.05, ** *p* < 0.01 vs. saline RA; ^#^ *p* < 0.05, ^##^ *p* < 0.01 vs. saline 50% O_2_; ^†^ *p* < 0.05, ^‡^ *p* < 0.01 vs. saline IH by ANOVA; ^§^ *p* < 0.05, ^§§^ *p* < 0.01 vs. bumetanide RA by unpaired *t*-test). RA (room air); O_2_ (oxygen); IH (intermittent hypoxia).

**Table 3:** Retinal Morphometry at P21.

Morphometric Parameters	Saline RA	Bumetanide RA	Saline 50% O_2_	Bumetanide 50% O_2_	Saline IH	Bumetanide IH
Tortuosity Index	1.00 ± 0.01	1.02 ± 0.03	1.15 ± 0.01 **	1.04 ± 0.04 ^‡^	1.2 ± 0.01 **	1.04 ± 0.04 ^§§^
Diameter of Arteries	36.6 ± 0.79	31.5 ± 3.3	39.7 ± 1.0 *	32.4 ± 0.80 ^‡^	27.5 ± 0.88 **	28.1 ± 0.85
Diameter of Veins (µm)	45.2 ± 0.99	52.10 ± 4.5	23.1 ± 0.76 **	42.1 ± 1.6 ^#,‡^	43.8 ± 1.2	38.6 ± 1.3 ^##,§§^
Number of Cells in NFL/GCL (µm)	210.0 ± 13.6	330.1 ± 5.4 **	424.8 ± 47.4 *	344.4 ± 19.6 ^‡^	994.7 ± 93.4 **	571.5 ± 49.3 ^##,§§^
Total Retinal Thickness (µm)	263.5 ± 4.3	321.3 ± 7.0 **	364.0 ± 3.8 **	326.0 ± 8.7 ^‡^	441.8 ± 14.6 **	366.9 ± 7.9 ^##,§§^
NFL/GCL Thickness (µm)	42.4 ± 1.4	55.8 ± 2.2 **	61.8 ± 2.9 *	57.3 ± 2.0	117.1 ± 8.8 **	64.4 ± 1.9 ^##,§§^
IPL Thickness (µm)	56.3 ± 0.88	77.2 ± 1.4 **	73.4 ± 3.3 **	64.7 ± 1.9 ^†^	63.8 ± 3.2 *	63.4 ± 2.4 ^##^
INL Thickness (µm)	48.4 ± 1.1	70.5 ± 2.0 **	70.5 ± 2.0 **	65.7 ± 1.4 ^‡^	78.1 ± 3.3 **	65.6 ± 1.9 ^##,§§^
ONL Thickness (µm)	67.5 ± 2.8	83.1 ± 1.8 **	102.6 ± 3.7 **	62.6 ± 1.7 ^‡^	110.8 ± 1.5 **	100.3 ± 1.7 ^##,§§^

Data are the mean ± SD (*n* = 24 measurements/group; * *p* < 0.05, ** *p* < 0.01 vs. saline RA; ^#^ *p*<0.05; ^##^ *p* < 0.01 vs. bumetanide RA; ^†^ *p* < 0.05, ^‡^ *p* < 0.01 vs. saline 50% O_2_; ^§^ *p* < 0.05, ^§§^ *p* < 0.01 vs. Saline IH. P21 (21 days postnatal age); RA (room air); O_2_ (oxygen); IH (intermittent hypoxia); NFL/GCL (nerve fiber layer/ganglion cell layer); IPL (inner plexiform layer); INL (inner nuclear layer); ONL (outer nuclear layer). Data were analyzed using ANOVA with Dunnett’s multiple comparison post hoc test.

## Abbreviations

ADPase	Adenosine diphosphatase
ANOVA	Analysis of variance
AQPs	Aquaporins
ARVO ELISA GCL	Association for research in vision and ophthalmology Enzyme-linked immunosorbent assay Ganglion cell layer
GFAP	Glial fibrillary acidic protein
H&E IGF-I	Hematoxylin and eosin Insulin-like growth factor-1
IH	Intermittent hypoxia
INL	Inner nuclear layer
IP	Intraperitoneal
IPL	Inner plexiform layer
KCC2	K^+^Cl_2_ cotransporter isoform 2
NFL	Nerve fiber layer
NKCC1	Na^+^/K^+^/Cl^−^ cotransporter
O_2_ OIR	Oxygen Oxygen-induced retinopathy
ONL	Outer nuclear layer
OPL	Outer plexiform layer
P14	Postnatal day 14
P21	Postnatal day 21
P0 RA R/C	Postnatal age at birth Room air Rods and cones
ROP	Retinopathy of prematurity
SEM	Standard error of the mean
SPSS sVEGFR-1	Statistical package for social sciences Soluble vascular endothelial growth factor receptor-1
VEGF	Vascular endothelial growth factor
